# Functional compartmentalization in the hemocoel of insects

**DOI:** 10.1038/s41598-019-42504-3

**Published:** 2019-04-15

**Authors:** Hodjat Pendar, Jessica Aviles, Khaled Adjerid, Caroline Schoenewald, John J. Socha

**Affiliations:** 1 Department of Biomedical Engineering and Mechanics, Virginia Tech, Blacksburg, VA 24061 United States; 2 Department of Mechanical Engineering, Sharif University of Technology, Tehran, Iran; 3 Grado Department of Industrial and Systems Engineering, Virginia Tech, Blacksburg, VA 24061 United States

## Abstract

The insect circulatory system contains an open hemocoel, in which the mechanism of hemolymph flow control is ambiguous. As a continuous fluidic structure, this cavity should exhibit pressure changes that propagate quickly. Narrow-waisted insects create sustained pressure differences across segments, but their constricted waist provides an evident mechanism for compartmentalization. Insects with no obvious constrictions between segments may be capable of functionally compartmentalizing the body, which could explain complex hemolymph flows. Here, we test the hypothesis of functional compartmentalization by measuring pressures in a beetle and recording abdominal movements. We found that the pressure is indeed uniform within the abdomen and thorax, congruent with the predicted behavior of an open system. However, during some abdominal movements, pressures were on average 62% higher in the abdomen than in the thorax, suggesting that functional compartmentalization creates a gradient within the hemocoel. Synchrotron tomography and dissection show that the arthrodial membrane and thoracic muscles may contribute to this dynamic pressurization. Analysis of volume change suggests that the gut may play an important role in regulating pressure by translating between body segments. Overall, this study suggests that functional compartmentalization may provide an explanation for how fluid flows are managed in an open circulatory system.

## Introduction

Insects use multiple mechanisms to transport hemolymph, the circulatory fluid of invertebrates. The major driver of flow is understood to be the dorsal vessel, which consists of a posterior heart and anterior aorta, found in all insects^1,2^. Accessory pulsatile organs, which supplement flow into the legs, wings, and antennae in some species, also augment flow production^2–4^. Both the dorsal vessel and accessory pulsatile organs are muscular pumps, which can produce relatively fast flows^4^. Although these components have been relatively well studied, they represent only a small fraction of the volume of the circulatory system. When hemolymph leaves the dorsal vessel, it enters the hemocoel, the main body cavity. In contrast to the vascular portion, the hemocoel contains a multitude of non-circulatory components, such as tracheal tissue, digestive system, fat bodies, and reproductive anatomy. The hemolymph must move through interstices among these components with relatively slow flow speeds (compared to heart flows) and ill-defined pathways as a consequence of an open system. These features have led some investigators to postulate that circulation is poorly regulated in this part of the body^5^. However, little is known about how flows are produced and controlled in the hemocoel^2^.

One potential mechanism for creating flows in the hemocoel is through the action of the abdominal pump^6^. The abdomen acts as a pump using coordinated action of intersegmental and dorsoventral muscles, producing a volume contraction of the abdomen in the dorsoventral or cranial-caudal axis, with variation across taxa. The abdominal pump has most often been implicated as a mechanism of active respiration, but abdominal pumping does not always result in respiratory airflow. For example, in a study of *Zophobas morio* beetle larvae^7^, more than half (63.7%) of the observed abdominal pumping events occurred while the spiracles were closed, resulting in no tracheal compressions producing advection and no external gas exchange. Similar results were found in studies of adult *Schistocerca gregaria* grasshoppers^8^ and *Bombus terrestris* bumblebees^9^, in which individuals exhibited periods of abdominal pumping with no CO_2_ emission ranging in duration from 2 to 30 minutes. These behaviors have been hypothesized to serve to mix gases within the tracheal system or to preserve water^8^, but it has also been suggested that abdominal pumping is used for other non-respiratory functions^10,11^. Abdominal pumping has been correlated with heartbeat patterns in some pupae^6,12–16^ and is well known to influence hemolymph pressure^7,10,11,17,18^ in many species, suggesting that it can have a circulatory role.

For the abdominal pump to create hemolymph flows within the hemocoel, it must create a pressure gradient. Measurements from a single location in the body have shown the existence of pressure pulsations ranging in amplitude from hundreds of pascals to 2.7 kPa, and such pulses may be correlated with abdominal pumping^10,17,19,20^. Knowledge of pressures at multiple locations would reveal pressure gradients, but such data are rare. Recordings taken separately in the head and abdomen of lepidopteran pupae (*Sarcophaga crassipalpis*) suggest that small differences (up to 10 Pa) may be created within the hemocoel^21^. Simultaneous measurement from the thorax and abdomen of a single blowfly specimen (*Calliphora vicina*) revealed much larger pressure differences, up to 700 Pa^19^. These large pressure differences resulted from tidal movement of the hemolymph between segments, facilitated by reversals in direction of flow through the dorsal vessel. This tidal behavior, well known in *Diptera*, *Hymenoptera*, and *Lepidoptera*^16,19,22–25^, likely depends on the presence of a constriction in the body between abdomen and thorax, which includes internal anatomy such as large air sacs and viscera that help to separate or compartmentalize these regions^26^. For insects that lack obvious constrictions between segments, the properties of an open hemocoel would suggest that pressure gradients created by the abdominal pump should be small or negligible^18^.

However, it is possible that insects that lack an external narrowing at the waist can exhibit internal partitioning by virtue of tightly packed internal anatomy, or by movement of tissues and organs to functionally compartmentalize regions within the body. For example, preliminary data from grasshoppers suggest that air sacs in the head and abdomen behave differently depending on sedation^27^. In sedated specimens held vertically and viewed with x-rays, air sacs at the bottom of the animal were found to be compressed whereas those at the top were inflated. When the specimen was flipped, the same compression pattern was found at the top and bottom, despite the head and tail reversing positions. Strikingly, this pattern disappeared when the specimen was not sedated—the air sac status did not change with orientation, suggesting that grasshoppers may actively regulate their internal pressure between different parts of the hemocoel. If so, this result suggests that the hemocoel may not always behave as an ideal open system, in which changes in fluidic pressure should propagate at the speed of sound; instead, it may exhibit some form of compartmentalization, in which pressures can be fully or partly isolated across the system. However, direct observations of hemolymph pressure are needed to test this hypothesis.

Here we broadly ask, is hemolymph pressure actively regulated within the hemocoel in insects? And, does the abdominal pump create pressure differences within the hemocoel, capable of driving circulatory flows? If the hemocoel behaves as a single fluidic compartment, then pressure differences within it should be small or negligible. Conversely, if the insect possesses morphological or dynamic mechanisms to regulate pressure, then differences may be substantial. We address these hypotheses by measuring hemolymph pressure in two locations in the beetle *Zophobas morio*, which does not possess an obvious constriction between the thorax and abdomen. In addition, we measured pressures while recording the displacement of the abdominal pump, and conducted a preliminary morphological investigation using dissection and x-ray tomographic imaging. This study investigates how insects manage fluidic pressure, providing new insight into the suite of mechanisms available for regulating hemolymph flow in the hemocoel and throughout the body.

## Materials and Methods

### Animals

We used 35 adult tenebrionid beetles, *Zophobas morio* Fabricius, 1776 (Coleoptera: Tenebrionidae) with a mass of m = 546 ± 35 mg (mean ± SD). The beetles were purchased from a vendor (Carolina Biology Supply, NC, USA), maintained at room temperature (20–23 °C) in a terrarium containing a mixture of sand and soil, and fed a diet of bran meal and water *ad libitum*. Both males and females were used, but were not identified. Separate specimens were used for pressure measurement (N = 20), x-ray tomography (N = 5), anatomical dissection (N = 6), and to measure the abdominal volume displacement and heart activity (N = 4).

### Pressure measurement

Two 420-μm Fabry-Perot fiber optic pressure sensors (Preclin 420, Samba Sensors, Gothenburg, Sweden) were used to simultaneously record hemolymph pressure in two locations in the hemocoel. To examine the compartmentalization hypothesis, we simultaneously recorded pressure in the abdomen and the thorax. In additional trials, we placed both sensors in the abdomen or the thorax to test the possibility of compartmentalization within a body segment. The sensors were calibrated using a water column before the experiments. During experiments, hemolymph gradually coagulated around the sensor tip. Because the tip of the sensors were immersed in the hemolymph, the coagulation rate was very slow. Testing of the sensors immediately after two trials (by re-running the calibration procedure) showed ±5% error due to the slight coagulation of the hemolymph, verified by inspection of the sensor tip under microscope. The signals of the pressure sensors were converted to analog signals using a signal conditioner (Samba 202, Samba Sensors, Gothenburg, Sweden) and data were recorded on a computer using an analog to digital converter (NI-9215, National Instruments, Austin, TX, USA).

**Figure 1:**
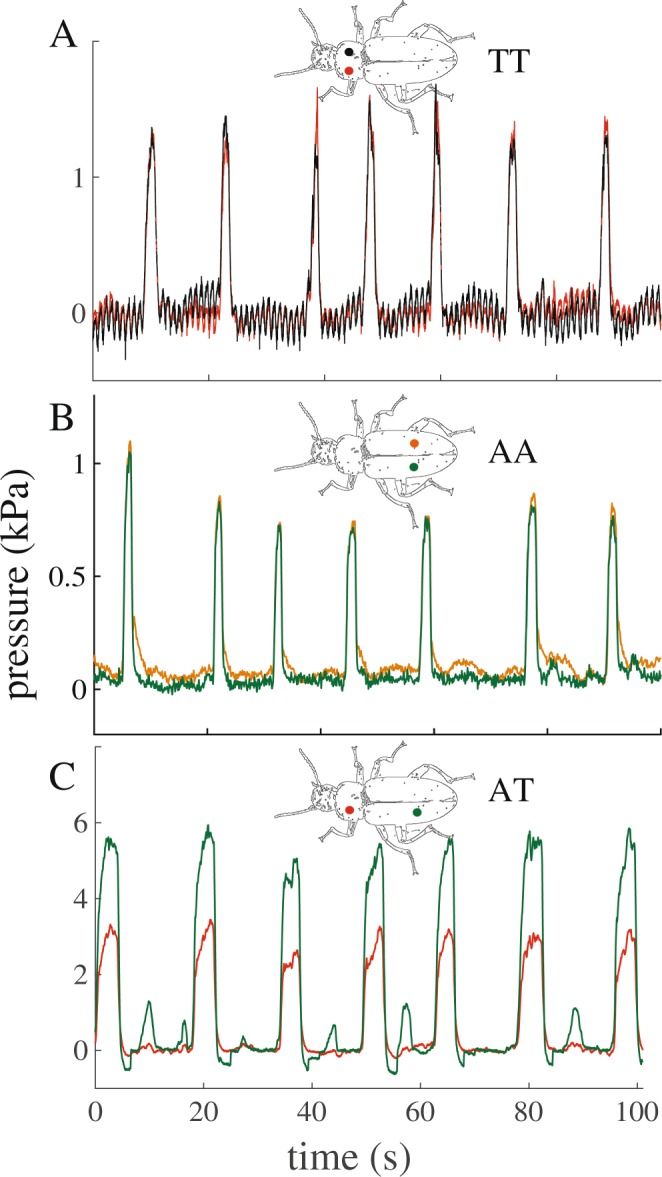
Representative hemolymph pressure traces in the abdomen and thorax of the beetle *Zophobas morio*. The hemolymph pressure differs substantially only between the abdomen and thorax, and not within the same segment. (**A**) TT (thorax-thorax): left and right sides of thorax. The small peaks likely represent the heartbeat. (**B**) AA (abdomen-abdomen): left and right side of the abdomen. (**C**) AT (abdomen-thorax): abdomen and thorax. Note also the small pulses in the abdomen without a corresponding pulse in the thorax.

To prepare for sensor implantation, beetles were cold-anesthetized at 3 °C. Their legs, body, and antennae were secured using adhesive putty (Scotch adhesive putty, 3 M, Minnesota, USA) to prevent the animal movement during the pressure recording. The elytra and soft wings were secured to the side with insect pins to provide access to the soft cuticle of the abdomen without breaching the hemocoel. In each trial, two sensors were inserted into two locations in one of three combinations (Fig. 1): abdomen-thorax (AT; N = 10), thorax-thorax (TT; N = 4), or abdomen-abdomen (AA; N = 6). To insert a sensor into the abdomen, a small hole was made with the sharp tip of a dissection probe in the second or third tergite on the left or right side about 2 mm off-center of the cranial-caudal axis, to avoid the dorsal vessel. The sensor was inserted to a depth of 1–3 mm and oriented parallel to the heart. To insert a sensor into the prothorax, a hole was made using a drill and bit (0.5 mm) on the left or right side, approximately 1.5 mm from the dorsal vessel. The sensor was then inserted to a depth of 0.5–1.5 mm into the body. For trials with two sensors in the same segment, the sensors were placed at opposing locations across the cranial-caudal axis. Pressures were recorded continuously for an average of 18 hours at a sampling rate of 100 Hz. All trials were conducted at room temperature (20–23 °C).

### Data analysis

**Figure 2:**
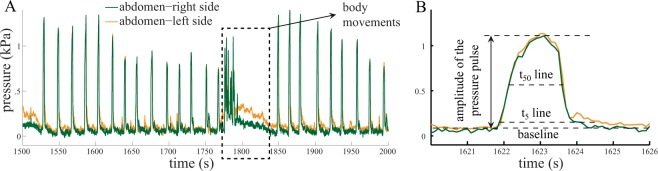
Sample recorded pressures from the left (L) and right (R) sides of abdomen. (**A**) The recorded pressure from different points of the abdomen show rhythmic patterns of pressure in different points of the abdomen. However, when the animal moves, a non-rhythmic pattern is generated, which was excluded from analysis. (**B**) The t_X_-line is a horizontal line that is X% of the pressure amplitude above the base line. Because the baseline of the signals were noisy, we could not accurately determine the start and end of the pressure pulses, and instead used the t_50_ line to quantify the duration of each pulse.

Pressure data were analyzed with a custom code using MATLAB software (MATLAB R2013a, MathWorks, Inc, USA). The code uses a simple moving average method with a window size of 11 to filter out high-frequency noise, which replaces each data point with the average of that point and 10 points around it. The baseline of the hemolymph pressure at times changed due to slow drift in the pressure sensors or a shift in the beetle’s body posture. To account for shifts in the baseline of the pressure signal, we determined a representative value for the baseline pressure every 5 minutes, using an average of the lowest 5% of the pressure data points in that interval, which encompasses most of the data points in the baseline. Animals occasionally and slightly moved their body. During these movements, the hemolymph pressure changed abruptly, which resulted in a different pattern than the regular pressure pulses. These pressure changes were usually quick and not periodic (Fig. 2). We eliminated these pressure pulses from analysis by considering only ‘regular’ pressure pulses. We define a regular pulse as one in which the magnitude was greater than a threshold *P*_*o*_ and the duration was greater than 300 ms. *P*_*o*_ was between 0.3 and 0.5 kPa, with the value for each specimen chosen to eliminate random movements. If a pulse met these criteria, then data during this time period were analyzed for each pressure sensor.

The amplitude of each pressure pulse was calculated by subtracting the pressure peak from the baseline (Fig. 2B). To determine the start and end of each pressure pulse, we identified the t_95_ line for each pulse, which is the horizontal line encompassing 95% of the pulse values above the baseline; the start time and end times are defined as the intersection of this line with the pressure trace. However, the filtering does not remove all of the noise from the baseline, and finding the exact start and end of the individual pressure pulses using t_5_-line was not very accurate (Fig. 2B). This issue becomes more important when the start and end of two pressure pulses in two different parts of the body are compared. Therefore, for comparisons of timing variables, we considered the time when the pressure pulses reached to 50% of their amplitude (using t_50_-line, Fig. 2B).

We also observed some isolated pressure pulses in the abdomen or in the thorax that were not associated with pressure pulses in the other location. To identify such pulses, we divided the pressure data into three categories based on the following magnitude criteria: (1) *P*_*A*_ > *P*_*o*_ and *P*_*T*_ > *P*_*o*_, indicating relatively large abdominal and thoracic pulses, where *P*_*A*_ and *P*_*T*_ are the pressures of the abdomen and thorax, respectively; (2) *P*_*A*_ > *P*_*o*_ and *P*_*T*_ > *P*_*o*_, indicating an abdominal pulse with almost no corresponding thoracic pulse; and (3) *P*_*A*_ > *P*_*o*_ and *P*_*T*_ > *P*_*o*_, indicating a thoracic pulse with no corresponding abdominal pulse.

**Figure 3:**
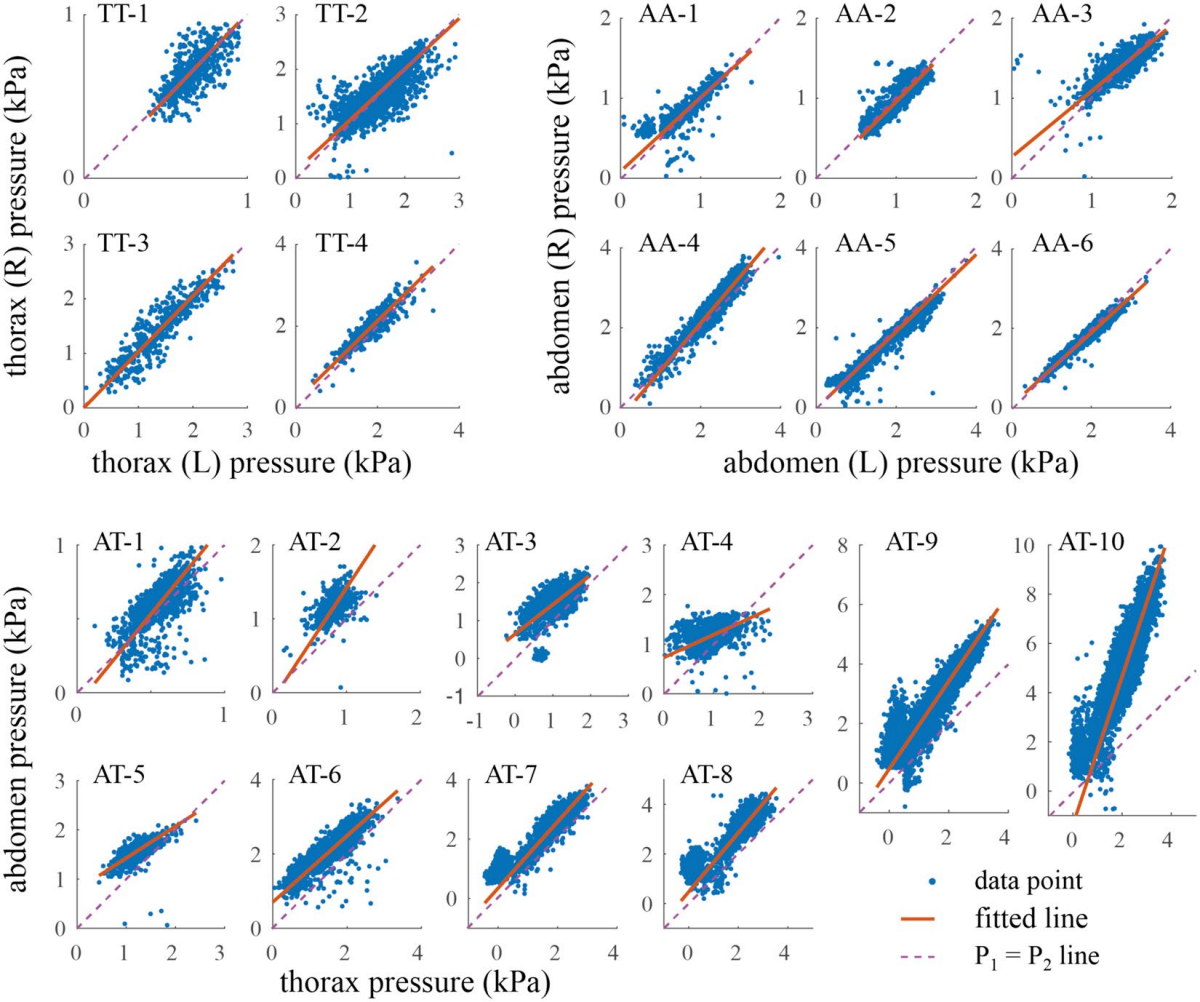
Comparison of the magnitude of the pressure pulses in the two simultaneously recorded locations. TT: thorax-thorax, AA: abdomen-abdomen, AT: abdomen-thorax; numbers indicate the individual beetle specimens. A line was fitted to the data points at each trial using the Deming method and compared with the P_1_ = P_2_ line, which indicates a uniform pressure distribution.

To compare concurrent pressure peaks at the two locations, corresponding pressure pulse magnitudes were plotted against each other for each specimen (Fig. 3). Because the pressure magnitudes from both sensors exhibit uncertainty, we used the Deming regression method^28^ to find the line of best fit for each data set. To fit a line to the data points of the abdomen-thorax trials, we only considered pulses in which both pressures were high (*P*_*A*_ > *P*_*o*_ and *P*_*T*_ > *P*_*o*_). To investigate the uniformity of the pressure in different points of the body, we calculated the distance of the pressure data points from the line *P*_1_ = *P*_2_ and normalized it by the distance of each data point from the origin. We calculated the mean and confidence interval of these normalized deviations from the *P*_1_ = *P*_2_ line (p = 0.05). If the confidence interval remained within the bounds of the sensor error (5%), we assumed the pressures to be equal; otherwise, we considered them as different.

### Abdominal pumping and heart activity

In addition to measuring the hemolymph pressure in the abdomen, we recorded the movement of the abdomen for 30 minutes from the side (N = 4) with a video camera (NEX-VG10, Sony) at 30 frames per second. In two specimens, we recorded the pressure in both abdomen and thorax; in the other two, we recorded the pressure in the thorax only. A flashing LED light was used to synchronize the video with the pressure signals. We used a custom MATLAB code to track 115 equally spaced points along the mid-tergites directly above the heart to determine the dorso-ventral displacement of the abdomen (N = 4). We assumed that the maximum displacement occurred at the midline of the abdomen and the minimum displacement occurred at the sides of the abdomen, where the abdominal cuticle merges with the exoskeleton. The volume change of the abdomen was determined by integrating the dorso-ventral displacement over the abdominal surface.

By opening the hard elytra and displacing the soft wings, the movement of the heart can be seen through the transparent cuticle of the abdomen. Specifically, the tissues adjacent to the heart were observed to move rhythmically, and we assumed that this movement reflected the movement of the heart. We recorded this activity with a video camera in the beetles for 5 minutes (N = 4). To determine the heartbeat frequency, we counted the number of beats per 30 seconds for each specimen. To estimate the velocity of the wave propagation along the heart, we determined the time for the wave to propagate per distance by analyzing the movies frame by frame using Final Cut Pro software (v7.0.3, Apple Computer Company, CA, USA), measuring 10 heartbeats from each specimen.

### Dissections

We performed dissections to determine the morphology of the internal cuticle between the abdomen and thorax and to trace the path of the gut from the thorax to the abdomen (N = 6). Adult beetles were sacrificed with fumes of ethyl acetate and the elytra and soft wings were removed. Animals were manually bent along the dorsal surface, opening the ventral cuticle between the abdomen and thorax, to directly observe the internal components. We also cut the body in different locations of the thorax to observe the muscles and the gut.

### X-ray tomography

To determine the three-dimensional internal anatomy of the beetles, we used synchrotron x-ray phase-contrast micro-computed tomographic (SR-µCT) imaging at beamline 2-BM at the Advanced Photon Source at Argonne National Laboratory. Because we aimed to maintain the internal morphology in its unaltered state, animals were imaged without fixatives or staining, following previously developed methods^29^. Beetles (N = 5) were first sacrificed using fumes of ethyl acetate, and then mounted in polyimide tubing (Kapton, Dupont, Wilmington, DE, USA). They were then placed in 4 °C refrigeration for two hours to allow residual internal movement to cease, and then rewarmed to room temperature for 30 minutes prior to imaging. Images were recorded with an exposure time of 7 s using a 2048 × 2048 px cooled-CCD sensor (CoolSNAP, Photometrics). The raw projections were taken every 0.125° as the sample was rotated over 180°. The x-ray beam supplied monochromatic light with an energy of 15 kEv, with a sample-to-detector distance of 30 mm. Avizo 3D rendering software (FEI Company, Berlin, Germany) was used to segment the 3D anatomy.

To measure the tracheal volume within the entire body, we used a custom MATLAB code to segment the tracheal system in the tomographic images from one specimen. Using this semiautomatic code, we analyzed 3,700 images per specimen to select and separate the tracheal tubes with the width of more than 5 µm.

## Results

Hemolymph pressure pulses occurred periodically in both the abdomen and the thorax. Pressure pulse amplitudes ranged from 0.54 to 4.62 kPa. The duration of pulses ranged from 0.9 to 3.35 s, with a mean of 1.78 ± 0.75 s and frequency ranging between 2.3 and 12.9 pulses per minute (0.09 ± 0.05 Hz; Table 1). The average time for the rise of the pressure from t_10_ to t_90_ was 0.44 ± 0.28 s.

**Table 1:** Summary of hemolymph pressure in the abdomen and thorax of all specimens. CI: confidence interval (p = 0.05).

Type of trial	Specimen ID	Number of pulses	P_1_ (kPa)	P_2_ (kPa)	Pulse duration (s)	CI of t_50_-start difference (ms)		CI of t_50_-end difference (ms)		CI of the normalized deviation from *P*_1_ = *P*_2_ (percentage)		Slope	freq. (1/min)
TT	1	896	1.50 ± 0.34	1.54 ± 0.32	1.23	−14	−7	−5	−1	1.16	1.64	0.93	2.74
	2	2350	1.84 ± 0.44	1.97 ± 0.43	1.48	−17	−3	−26	−17	3.16	4.17	0.98	2.35
	3	7026	0.66 ± 0.11	0.65 ± 0.13	3.35	−20	25	5	59	−1.72	−0.72	1.05	2.87
	4	3870	1.39 ± 0.52	1.43 ± 0.53	0.89	4	10	−2	4	0.5	2.43	1.03	8.16
AA	5	1669	0.75 ± 0.27	0.80 ± 0.22	1.51	−39	−28	−29	−20	3.72	5.61	0.92	2.89
	6	5377	1.00 ± 0.21	0.96 ± 0.21	2.73	2	8	−51	−46	−2.34	−1.93	1	2.87
	7	3188	1.85 ± 0.56	1.76 ± 0.54	1.25	1	5	−9	−6	−2.59	−2.09	0.96	9.43
	8	458	2.24 ± 0.39	2.40 ± 0.46	2.27	−78	−69	−66	−60	3.11	3.4	1.18	2.86
	9	695	1.36 ± 0.21	1.39 ± 0.17	2.62	11	20	−32	−23	0.97	1.54	0.82	6.73
	10	407	2.00 ± 0.30	1.90 ± 0.27	1.95	−8	−4	1	2	−2.78	−2.65	0.91	3.73
AT	11	1112	0.83 ± 0.15	1.16 ± 0.18	1.56	10	38	6	14	16	17.57	1.51	5.86
	12	668	1.22 ± 0.31	1.56 ± 0.25	0.93	−12	−5	−18	−13	11.63	13.51	0.64	12.9
	13	5446	1.58 ± 0.52	2.12 ± 0.46	1.36	−22	−7	−42	−34	15.42	16.34	0.89	2.98
	14	13540	1.18 ± 0.93	1.91 ± 0.78	1.9	0	5	−4	0	31.69	32.72	1.12	8.23
	15	5332	1.41 ± 0.84	2.66 ± 1.10	1.39	13	25	−23	−16	32.51	33.08	1.47	2.81
	16	2409	0.54 ± 0.11	0.59 ± 0.14	1.2	11	47	5	21	2.72	3.91	1.22	4.07
	17	9476	1.92 ± 0.80	4.62 ± 1.88	2.12	−30	−18	−44	−31	37.77	38.44	3.01	6.19
	18	7472	1.56 ± 1.03	2.62 ± 0.87	2.16	−10	−4	−47	−40	30.82	31.88	1.19	7.26
	19	1297	1.05 ± 0.40	1.45 ± 0.39	1.76	−52	−7	−109	−56	15.19	17.2	0.78	7.36
	20	1723	0.95 ± 0.31	1.17 ± 0.22	0.97	10	67	−109	−75	10.24	12.16	0.46	8.36

The average difference between the left and right thoracic pressure pulses was 0.05 ± 0.06 kPa, and the average difference of the t_50_-start, t_50_-end, and peak of the two pressure pulses were 2 ± 8 ms, 75 ± 160 ms, and 4 ± 13 ms, respectively (Table 2). The average slope of the regression lines for the pressure measurement points across beetles was 0.99 ± 0.05 (p < 0.05, Fig. 3). The upper and lower bounds of the confidence interval of the normalized deviation from *P*_1_ = *P*_2_ line (p = 0.05) for all the trials was in the range of the sensor error (5% error).

**Table 2:** Summary of differences in timing and pressure magnitudes of two simultaneously recorded pressures from two points of the body.

Probe location	N	n	t_50_ start diff. (ms)	t_50_ end diff. (ms)	peak diff. (ms)	pressure diff. (Pa)
thorax-thorax (TT)	4	4748	2 ± 8	75 ± 160	4 ± 13	48 ± 60
abdomen-abdomen (AA)	6	21188	14 ± 32	27 ± 24	33 ± 31	0 ± 99
abdomen-thorax (AT)	10	59247	−2 ± 22	30 ± 30	17 ± 36	760 ± 778

N: number of specimens, n: total number of pulses, t_50_ start/end: time points at which pressure pulse is at 50% of its peak magnitude when increasing or decreasing. This table shows the t_50_ start/end times between the pressure sensor signals are almost identical for all three sensor placement configurations. However, while these coinciding pressure pulses in the same segments (TT, AT) were similar in magnitude, coinciding pressure pulse magnitudes between segments (AT) were different in magnitude.

The average difference between the left and right abdominal pressure measurements was 0.00 ± 0.1 kPa, and the difference of the t_50_-start, t_50_-end, and peak of the pressure pulses were 14 ± 32 ms, 27 ± 24 ms, and 33 ± 31 ms, respectively (Table 2). The average slope of the regression lines for the pressure measurement points across beetles was 0.97 ± 0.12 (p < 0.05, Fig. 3). The confidence interval (p = 0.05) of the normalized deviation from *P*_1_ = *P*_2_ line for all trials was within the sensor error range 5%, except in one trial, in which the upper bound of the error (5.9%) was slightly above the sensor-error range.

When comparing pressure pulses between the abdominal and thoracic segments, the variation was higher than in the previous same-segment comparisons (Fig. 3). The average pressure was 1.22 ± 0.4 kPa in the thorax and 1.98 ± 1.3 kPa in the abdomen across all specimens. The average pressure difference between the abdomen and thorax was 0.76 ± 0.78 kPa (abdominal pressure minus thoracic pressure), and the difference between the t_50_-start of the two pressure pulses was only −2 ± 22 ms, indicating that the pulses started together (Table 2). The normalized deviation from *P*_1_ = *P*_2_ line was above the range of the sensor-error in all the trials except in one (2.7%-3.9%).

**Figure 4:**
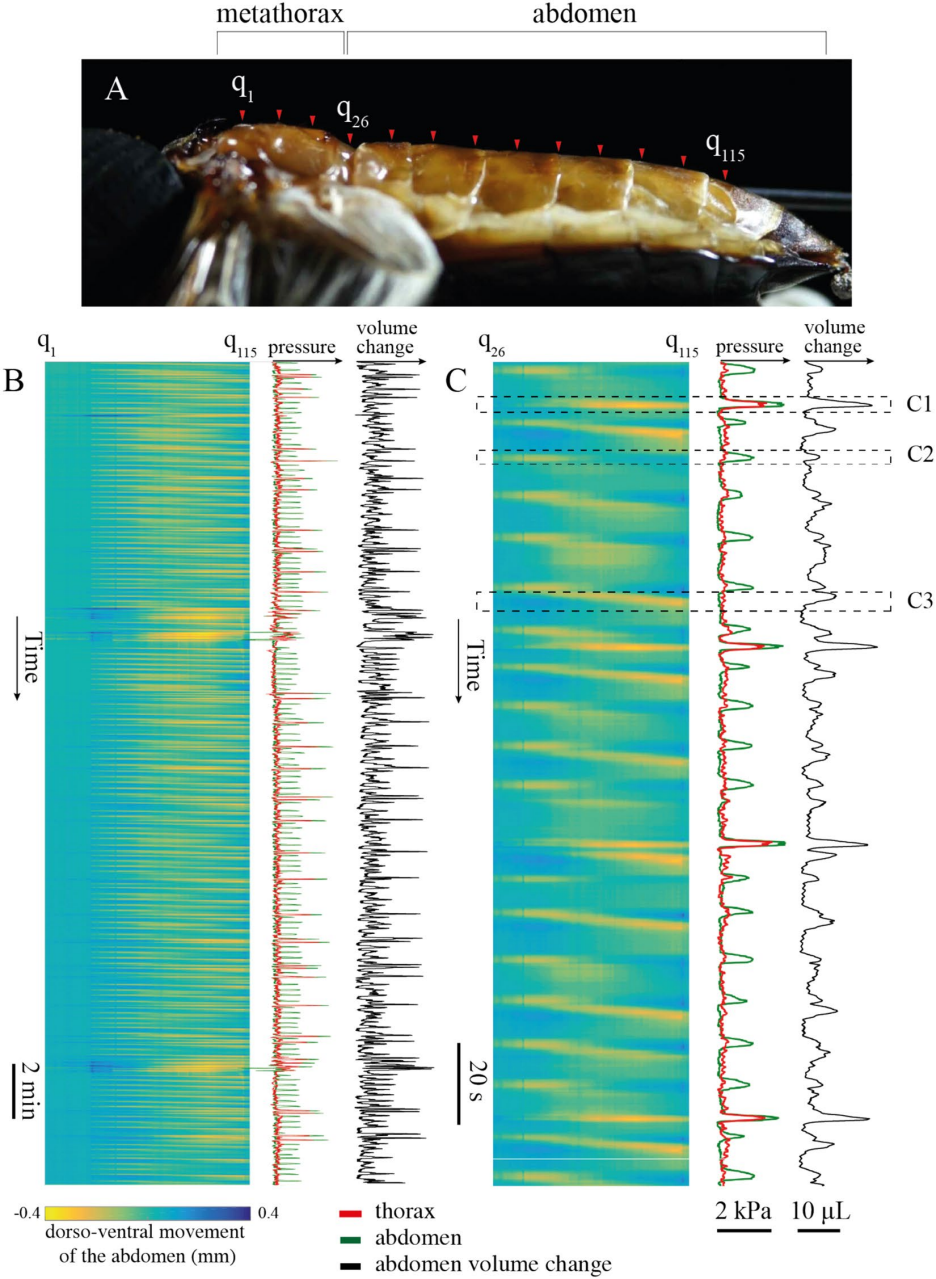
Abdominal movements versus pressure change in the abdomen and thorax. (**A**) 115 equally spaced points (q_1_ to q_115_), representing 25 on the metathorax and 90 on the abdomen, were tracked frame by frame. (**B**,**C**) Each pressure pulse in the abdomen and thorax was coincident with a movement of the abdomen. When all the tergites compressed simultaneously (C1), the pressure in both the abdomen and thorax increased. When the first two tergites compressed ventrally (C2), only the pressure in the abdomen increased, without a significant change in the pressure of the thorax. No significant pressure change was observed when a peristaltic wave propagated posteriorly (C3).

Most pressure pulses in the abdomen coincided with large pressure pulses in the thorax. However, 32.9% of the abdominal pressure pulses coincided with small or negative pressure changes in the thorax (Fig. 3). Similarly, 3.1% of the large pressure pulses in thorax coincided with small pressure changes in the abdomen. Image analysis of abdominal movement revealed multiple types of movement (Fig. 4). In the main observed behavior, all tergites compress dorso-ventrally together, which we defined as true abdominal pumping (Fig. 4-C1). This behavior coincided with a large pressure pulse in both abdomen and thorax. The second type of observed abdominal behavior is a peristaltic motion, in which a wave with the amplitude of 0.15 ± 0.08 mm and speed of 9.7 ± 2.5 mm/s propagated along the length of the abdomen. This type of movement did not coincide with pressure pulses in the abdomen or the thorax (Fig. 4-C3). In the third observed behavior, the first two to three segments of the abdomen slightly compressed and the other segments expanded, and vice versa (Fig. 4-C2). This movement coincided with small pressure peaks in the abdomen but no pulses in thoracic pressure. We define this type of abdominal movement as a ‘pinching’ motion. The estimated volume displacement of the abdomen during an abdominal pumping event for 4 recorded specimens (m = 594 ± 58 mg) was 19.7 ± 3 µL, which is equivalent to 0.033 L/kg or 3.2% of the body volume, assuming 2.5% of the body volume is trachea (see next paragraph). The sternite of these beetles is not very flexible and unlike in insects such as mosquitoes, which contract the ventral abdomen^30^, we did not notice any considerable contraction in the ventral side of the abdomen in *Z. morio*.

**Figure 5:**
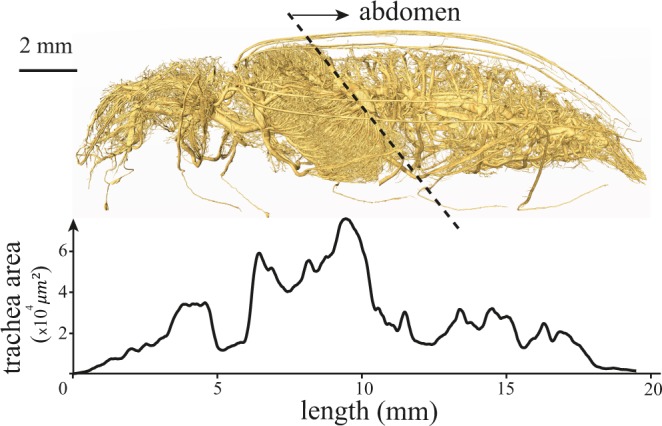
Distribution of the tracheae across the body of the beetle *Zophobas morio*. This volumetric analysis was based on 3700 tomographic images from one individual, representing tracheae of diameter greater than 5 µm; smaller tracheae and tracheoles could not be resolved in the x-ray images and were not included. The volume of tracheae is greatest in the meso- and metathorax, where the flight muscles are concentrated. About 35% of the tracheal volume is in the abdomen.

Analysis of the tracheal system showed that the total tracheal volume is 14.7 µL (Fig. 5) for a 583 mg beetle (0.025 µL/mg). There are more tracheae in the meso- and metathorax, which are densely filled with flight muscles, and only 5.2 µL of the tracheae (35.4%) is in the abdomen. This analysis included only the tracheae with the diameter of more than 5 µm, so the true volume must be greater. (Based on an analysis of tracheae in stick insects^31^, this error may be on the order of 30%.) Assuming the density of the tissues is about the density of water, we can estimate that 2.5% of the body volume is the tracheal tubes.

The recorded videos of the heart activity showed the frequency and speed of the heart peristalsis are 1.0 ± 0.21 Hz and 24.1 ± 4.3 mm/s, respectively, with the frequency an order of magnitude greater than the frequency of abdominal pumping (0.09 ± 0.05 Hz).

**Figure 6:**
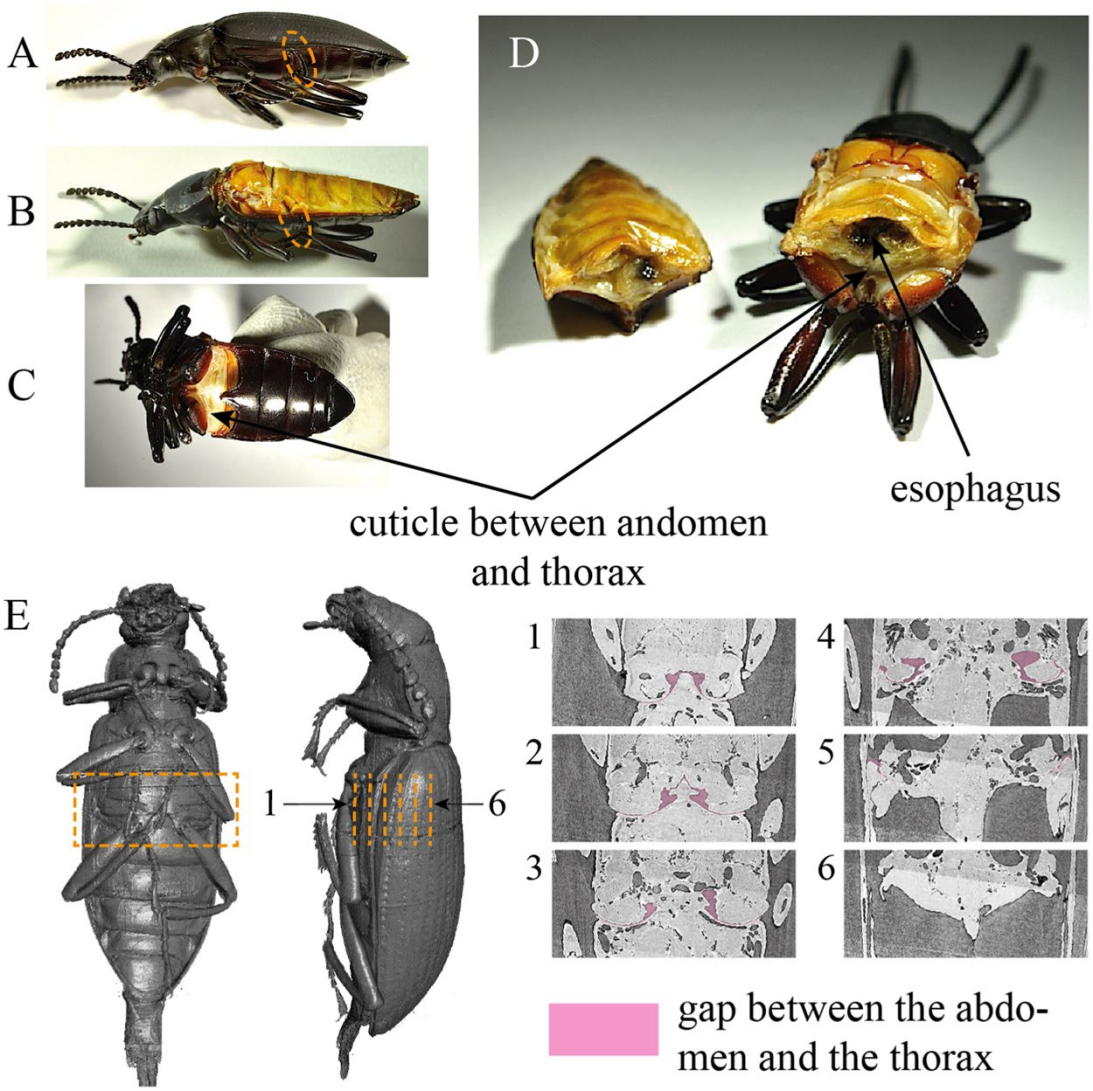
Morphology that may contribute to internal compartmentalization. The cuticle between the abdomen and thorax may function to isolate the thorax from the abdomen. (**A**) This cuticle is located posteriorly to the hind coxae, between the abdomen and thorax. (**B**) To observe this cuticle the wings were removed and (**C**) the beetle was bent dorsally. (**D**) Cutting the abdomen helps to see the cuticle more clearly. It also reveals a channel between the abdomen and thorax, which is filled with the esophagus. (**E**) This cuticle and the gap between the abdomen and thorax can be observed in tomographic images as well.

**Figure 7:**
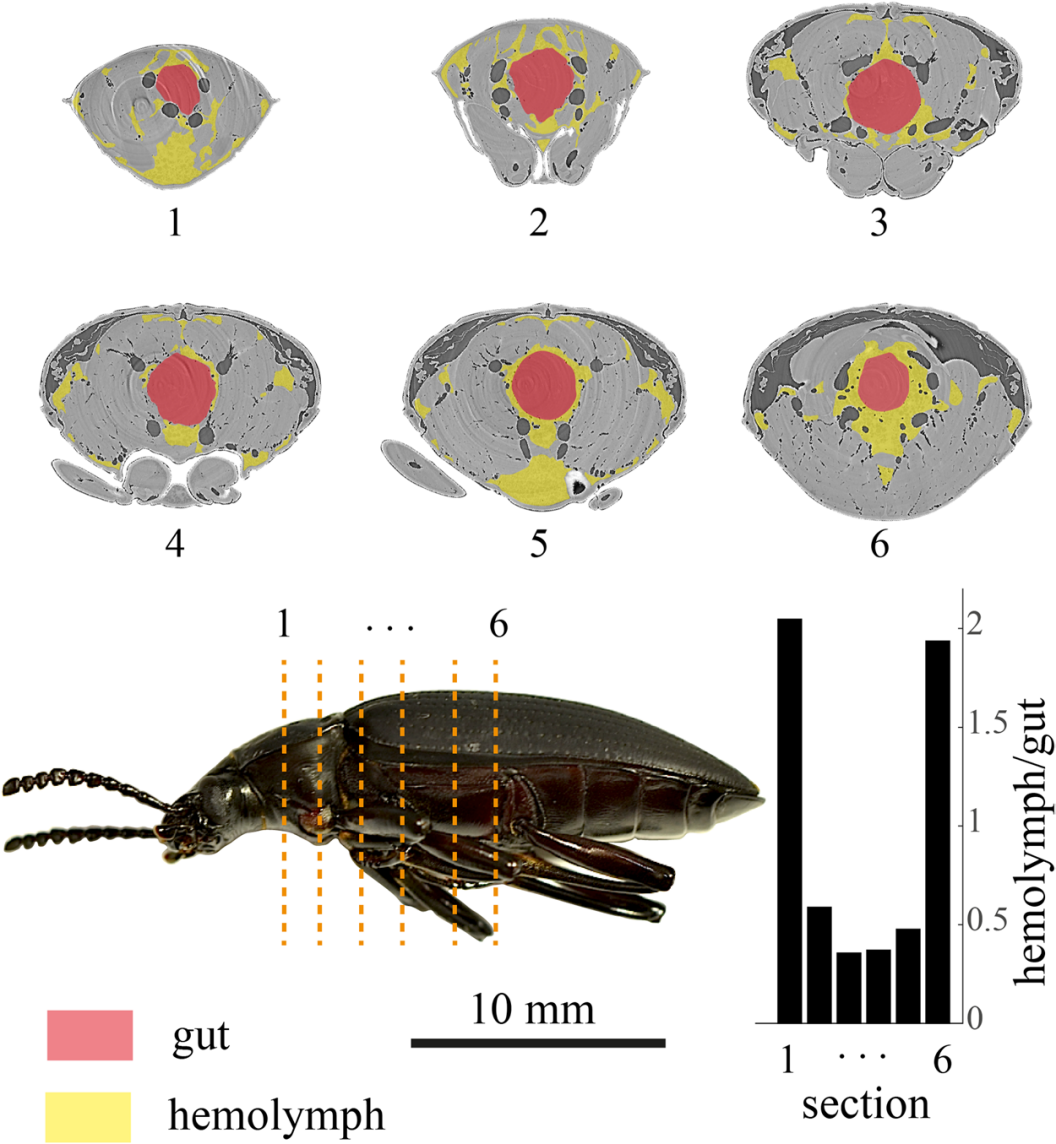
Large muscles in the thorax increase the impedance for hemolymph movement. The large gut of the beetles passes through the thoracic muscles. Any local increase in the volume of the gut would increase the impedance and possibly block the hemolymph passages. The area of the hemolymph around the gut is less than 35% of the gut area in some points of the thorax.

Dissections and tomographic imaging revealed a lack of anatomical compartments within the abdominal cavity (perivisceral sinus) and in the prothorax. However, we observed a section of cuticle between the abdomen and thorax (Fig. 6), that starts from the coxae of the hind legs on the thoracic side, and reaches diagonally to just below the middle of the abdomen. The tissue then angles ventrally on the abdominal side. Additionally, the tomographic images and dissections show a high density of muscles in the metathorax, with only a small channel that can act as a conduit for the alimentary canal, which is filled mostly by the esophagus (Fig. 7).

## Discussion

Insects have an open circulatory system, which may imply that the hemocoel behaves mechanically as a continuous vessel. However, to precisely manage the hemolymph movement within the hemocoel, insects may actively regulate the hemolymph pressure across their body cavity. Our pressure recordings in the thorax and abdomen of beetles enabled us to test this hypothesis. When the abdomen is at rest, the pressure is uniform between the abdomen and thorax, suggesting open fluidic communication between segments. During abdominal pumping, pressure pulses occur synchronously and are equal in magnitude at different locations in the abdomen or the thorax. This congruency in pressure indicates that, within both the abdomen and within the thorax, the hemocoel of these segments acts as a continuous vessel for the communication of hemolymph. However, this behavior changes when comparing pressure pulses across the abdomen and thorax. During abdominal pumping, hemolymph pressure increases synchronously across these segments, but the magnitude of the pressure pulses is different (Figs 1-C, 3 and 4-C1). This suggests that during abdominal pumping, fluidic communication of hemolymph becomes partially restricted between the abdomen and thorax, forming functional compartments. In other instances, pressure pulses occur separately in the thorax or abdomen, which is also congruent with compartmentalization. Overall, these results strongly suggest that insects can functionally compartmentalize their hemocoel, with each compartment experiencing different hemolymph pressures, a process that potentially contributes to regulating circulation of hemolymph.

In the specimens where both abdominal and thorax pressures were recorded, the average pressure in the abdomen was 62.3% higher than that of the thorax (Figs 1 and 3). The pressure difference remained elevated for an average of 1.5 s while the abdomen was compressing and then expanding. During this time, the direction of the pressure difference remained the same, indicating that this difference was not just a result of hemolymph movement. Furthermore, in the pinching motion, the pressure rose in the abdomen while the thoracic pressure was nearly zero or even negative in some cases (e.g., Fig. 3, AT7-10). Conversely, we also observed cases in which the pressure in the thorax increased without a significant change in the abdominal pressure (e.g., Fig. 3, AT3). During peristaltic motion, there were no pressure pulses in either the abdomen or thorax (e.g., Fig. 4-C3). In contrast to the prevailing understanding^2^, these results demonstrate that the insect’s hemocoel can be functionally compartmentalized, and pressure distribution in the hemocoel is not always uniform.

In the small open body cavity of insects, it is only possible for a large pressure difference to be held for extended periods of time if there are separated compartments. The compartments may be sealed off from one another, or there may be micro-scale openings between them in which fluid is allowed to continuously flow from high to low pressure. Investigating the relationship between the hemolymph pressure and the volume contraction of the abdomen may inform us of the mechanism behind compartmentalization and the nature of the connection between the compartments. The properties of hemolymph are close to water, and with the magnitude of the pressure pulses being relatively small (on the kPa scale), it is safe to assume that all the fluids inside the body are incompressible. Therefore, the change in volume of the abdomen during abdominal pumping should be equal to the sum of the volume of displaced fluids or tissues within the abdomen. There are three possibilities that can be considered to explain this abdominal volume change:

1. Compression of abdominal tracheae: In the behavior known as rhythmic tracheal compression, the insect compresses parts of tracheal system, which drives respiratory gases out of the body^32^ and must also correspondingly decrease the body volume. However, we estimated the volume of the tracheal tubes within the abdomen to be less than 0.9% of the body volume. Furthermore, the compressed volume must be some fraction of this value, because parts of the system could remain uncompressed^7^. The average volume contraction of the abdomen during abdominal pumping was calculated to be $\delta V=19.7\pm 3\,\mu L$ (3.2% of the body volume), a value that is much greater than the total estimated volume of the tracheae in the abdomen (5.2 µL). Therefore, the volume contraction of the abdomen cannot be accounted for by the compression of the tracheal system alone.
2. Hemolymph movement between compartments: During abdominal pumping, hemolymph pressure in the abdomen is on average higher than that observed in the thorax. Therefore, if these compartments are connected, hemolymph can move from the higher-pressure abdomen to the lower-pressure thorax. If this were true, then during the abdominal expansion, the hemolymph must move back to the abdomen, increasing the abdominal volume. However, we observed that, during abdominal expansion, the pressure in the abdomen remains higher than in the thorax (Figs 1 and 3). Therefore, during the expansion of the abdomen, there cannot be a pressure-driven fluid flow from the lower-pressure thorax to the higher-pressure abdomen. Therefore, pressure-driven hemolymph flow cannot be the means by which the volume contraction of the abdomen occurs.
3. Movement of visceral tissues, organs, or gut contents between the compartments: Visceral tissues and tracheae are located within the hemocoel and are not rigidly anchored in place. The largest of these organs are the esophagus and crop, which can move longitudinally along the body between the abdomen and thorax. Moreover, the esophagus and crop are flexible, muscular, have the ability to expand or contract, and move the gut contents back and forth. Although not previously noted, in some previously published x-ray videos of live insects the rhythmical movement of the gut and its content is noticeable^33^, with a similar rhythm of abdominal pumping^34–36^. The added volume to the thorax can be justified by the compression of the tracheal tubes in the thorax and head, and the expansion of pleural membrane between thoracial segments. The coordination of heart activity and gut movements is plausible given that a number of myotropic neuropeptides are known to modulate activity in both organs^37,38^. For example, proctolin increases the heart rate and also induces muscle contraction in the gut in some species^39,40^. The added volume to the thorax could be compensated by the compression of the tracheal tubes in the thorax and head, and the expansion of pleural membrane between body segments.

Although we have not directly observed the gut movement in this study, a sliding gut-piston has been observed at least once with x-rays^33^. Here, to show the possible consequence of gut motion, we provide a prediction of how such movement might affect volume change in *Zophobas*. We assume that the tracheal compression in the abdomen is negligible and that the volume contraction of the abdomen is equal to the volume displacement of the gut. From the anatomical dissections and x-ray images, we found the diameter of the esophagus to be about d = ~2.5–3 mm. Assuming that the anterior displacement of the gut or its content from the abdomen to the thorax is *δx*, then $\delta x=\frac{4}{\pi {d}^{2}}\delta V$, which leads to *δx* = ~2.7–4 mm. It is also possible to estimate how much of the diameter and cross-sectional area of the esophagus expands during a pump. Considering the esophagus to be a cylinder with a diameter of *d*, cross-sectional area of $A=\frac{1}{4}\pi {d}^{2}$, a constant length of *L* ≈ 12 *mm*, and a volume of *V* = *AL*, the volume change will be $\delta V=(\frac{1}{2}\pi Ld)\delta d=L\delta A$, where *δd* and *δA* are the average diameter change and cross-sectional area change of the esophagus, respectively. In this estimation, the volume expansion of the esophagus is almost equal to the volume contraction of the abdomen. Therefore, the change in the cross-sectional area of the esophagus during abdominal pumping is predicted to be *δA*/*A* = 23~33%, which is equivalent to *δd*/*d* = 11~16% increase in the diameter. This change in the cross-sectional area of the esophagus, which could happen due to the movement of the gut and gut contents, may explain how the abdominal and thorcic cavities become isolated from each other to experience different pressures during abdominal pumping. The expansion of the esophagus might block the narrow connections that pass through the thoracic muscles (Fig. 7), possibly isolating the abdominal and thoracic compartments during abdominal pumping. The preliminary assessment of the cross-sectional area of the gut and hemolymph shows that in some points of the thorax, the area of the hemolymph around the gut is less than 35% of the gut area (Fig. 7). This means that the expansion of the gut has the potential to block the path of the hemolymph in those locations.

It is beyond the scope of this study to investigate the physiological functions underlying compartmentalization. However, the potential of creating a pressure gradient across the body cavity that can induce hemolymph flow suggests that compartmentalization may contribute to the broader circulatory function within the insect, such as initiating heart activity^6,41–44^ and pumping hemolymph into the legs^10,11^.

If the hemocoel becomes compartmentalized during abdominal pumping, the gut might play a significant role in regulating the hemolymph circulation by translating between the compartments. The foregut, including the esophagus and crop, is large in comparison to the size of the body. Its motion between segments during abdominal pumping may be able to control the hemolymph pressure in the compartments by managing how much it moves between them (Fig. 7).

## Conclusions

This study demonstrates that large pressure differences can be created within the hemocoel of an insect that does not possess a narrow waist, and suggests that functional compartmentalization enables control of hemolymph flow aided by movement of tissues and organs between compartments. The combination of internal cuticles, dense flight muscles, tracheae, and gut may be functionally analogous to the constriction seen in insects with narrow waists. Grasshoppers have previously been predicted to functionally compartmentalize their hemocoel, suggested indirectly by imaging of the tracheal system^27^. Here, we provide direct evidence that hemolymph pressures can behave in an isolated or semi-isolated fashion, providing direct evidence of compartmentalization across the abdomen and thorax. New studies that probe compartmentalization across developmental stages and taxa will lead to a better understanding of how insects regulate circulation. In general, the circulatory systems of invertebrates are not very well understood and are described by some investigators as ‘sluggish’ or poorly regulated^5^, primarily because the system is open. The perivisceral sinus has at times been considered as a single column of hemolymph, which could lead to inaccurate interpretations about the general hemolymph flow within the animal^2,6,17,18^. The internal fluid movement within the hemocoel may be much more complicated than previously thought, with internal morphology and movement of organs contributing to precise hemolymph control and complex patterns of pressure.
